# Light After Darkness: A Case Report of Isolated Optic Perineuritis

**DOI:** 10.7759/cureus.55811

**Published:** 2024-03-08

**Authors:** Zulaikha Abdul Rahman, Shahidatul-Adha Mohamad, Hanisah Abdul Hamid

**Affiliations:** 1 Ophthalmology and Visual Science, School of Medical Sciences, Universiti Sains Malaysia, Kubang Kerian, MYS; 2 Ophthalmology, Hospital Sultan Abdul Halim, Sungai Petani, MYS; 3 Ophthalmology, Hospital Universiti Sains Malaysia, Kelantan, MYS

**Keywords:** neuromyelitis optica spectrum disorder, multiple sclerosis, corticosteroid, optic perineuritis, atypical optic neuritis

## Abstract

This is a report on remarkable visual recovery from blindness in a case of isolated optic perineuritis (OPN). A 68-year-old Chinese lady presented with a two-week history of progressive painless bilateral vision loss. Her vision was 6/18 on the right eye and no perception of light (NPL) on the left eye with positive relative afferent pupillary defect (RAPD). Fundus showed hyperaemic and swollen optic disc bilaterally. MRI of the brain and orbit revealed hyperintense periventricular white matter lesions, possibly early changes of multiple sclerosis (MS), and perineural enhancement of optic nerve bilaterally, consistent with OPN. All other investigations were negative. Intravenous methylprednisolone 1g/day for three days was started, followed with oral prednisolone, tapered in three months. At the third month of follow-up, her vision had improved to 6/12 on the left and 6/9 on the right. The hyperaemic and swollen disc has resolved. Intravenous megadose corticosteroid treatment is an effective first-line treatment for OPN.

## Introduction

Optic perineuritis (OPN) is a subtype of optic neuritis (ON) where the inflammatory process affects the meningeal sheath surrounding the optic nerve [1]. This is opposed to the ON where there is inflammation of the optic nerve axons [2]. OPN is a rare form of orbital inflammatory disease [3], in which the individuals with OPN frequently experience rapid vision loss that progresses over a span of weeks, accompanied by eye pain intensified by eye movement, which can be more severe or persistent compared to ON [2].

OPN may also be classified as atypical ON due to its non-demyelinating nature that can result from any cause such as an inflammatory, infectious, drug or autoimmune disorder [4]. The features of atypical ON include bilateral eye involvement, vision loss that progresses past two weeks in the absence of periocular pain, and vision that does not improve despite corticosteroid treatment [3]. In contrast, any visual impairment in OPN may have a rapid and dramatic respond to steroid [3]. In this article, we described a case report of remarkable improvement of severe vision loss in OPN.

## Case presentation

A 68-year-old Chinese lady presented with a two-week history of progressive painless bilateral loss of vision. She had no history of eye redness, discharge, photopsia, diplopia or prior ocular trauma. There was no neck stiffness, body weakness or other neurological deficit.

**Figure 1:**
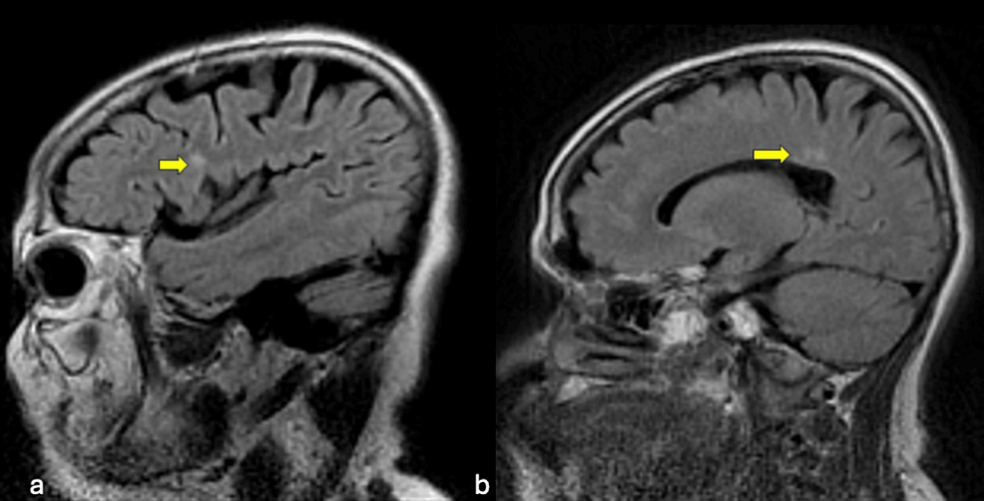
Sagittal T2 fluid attenuated inversion recovery (FLAIR) images (a & b) showed hyperintense periventricular white matter lesion (yellow arrow).

**Figure 2:**
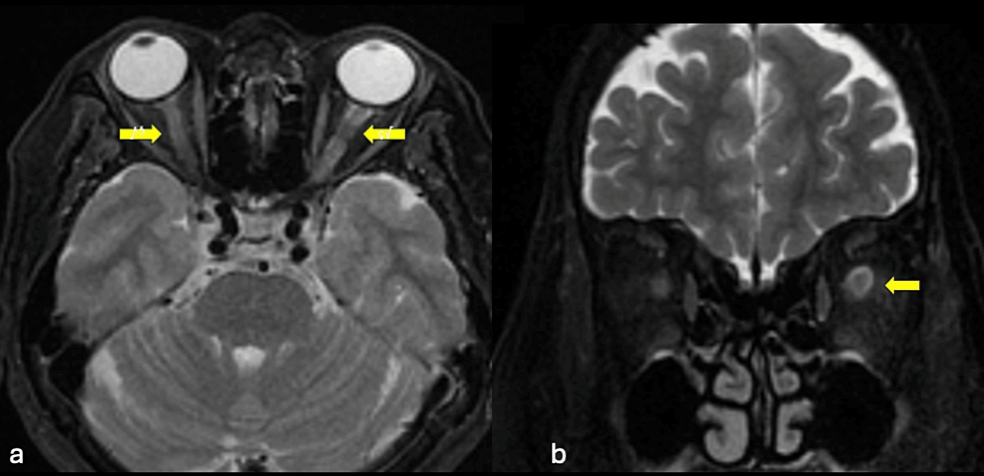
T2-weighted image axial view showed presence of bilateral tram-track sign (yellow arrow in a), and doughnut sign in the coronal view (yellow arrow in b).

On examination, her best-corrected vision was no perception of light (NPL) on the left eye and 6/18 on the right. Relative afferent pupillary defect (RAPD) was positive on the left. She failed to read Ishihara colour plate even with her right eye. Extraocular motility was full. Anterior segment examination was normal, and ocular media was clear for both eyes. Fundus examination showed hyperaemic and swollen optic disc bilaterally. The rest of the fundus examination was unremarkable. Connective tissue disease screenings as well as infective screening for tuberculosis, syphilis, herpes and cytomegalovirus were negative. Cerebrospinal fluid study was not done as patient refused for lumbar puncture. MRI of the brain revealed hyperintense periventricular white matter lesions possibly early changes of multiple sclerosis (MS) (Figure 1). MRI also showed a larger left optic nerve with surrounding fluid, consistent with left OPN (Figure 2).

She was treated with intravenous methylprednisolone 1g/day for three days, followed by oral prednisolone, gradually tapered in three months. At the third month of follow-up, her vision had improved to 6/12 on the left and 6/9 on the right, with improvement in brightness sensitivity, red desaturation and Ishihara color test on both eyes. The hyperaemic and swollen disc has resolved. The final diagnosis of isolated OPN was established after her negative anti-aquaporin-4 antibodies result.

## Discussion

OPN, also referred to as perioptic neuritis, is an uncommon type of orbital inflammatory condition characterized by inflammation of the optic nerve sheath, leading to significant thickening caused by nonspecific fibrosis [2]. Most OPN is categorized as primary, isolated and idiopathic in nature, while secondary OPN occurs following infections or autoimmune disease such as in antineutrophil cytoplasmic antibodies (ANCA)-associated vasculitis, sarcoidosis, syphilis and Crohn’s disease [3].

Initially, patients with OPN are prone to be misdiagnosed as ON due to the similarity in presenting signs of optic nerve dysfunction, such as diminished color perception and either swollen or seemingly normal optic nerve heads [2,5]. However, vision impairment in OPN has subacute onset, evolving over a period of several weeks, with severity varying from none to severe, and often characterized by descriptions of blurred vision, dimness, or the perception of splotches or "spots" in vision [3]. Patients diagnosed with OPN might exhibit paracentral or arcuate scotomas instead of the central scotoma typically observed in ON [6]. They might also have orbital signs such as diplopia, ptosis, ophthalmoparesis, and chemosis [6].

The diagnosis of OPN is on the basis of clinical and radiographic findings [3]. A distinctive MRI observation entails contrast enhancement of the optic nerve sheath while sparing the optic nerve itself [3]. In OPN, the perioptic enhancement manifests as a tram-track pattern in the axial view and a doughnut shape in the coronal view [2]. Nevertheless, the tram-track sign may also be present in other inflammatory or neoplastic conditions involving the optic nerve sheath, including optic nerve sheath meningioma, sarcoidosis, lymphoma, leukemia, orbital pseudotumor, perioptic hemorrhage, and metastasis [7]. Thus it is not specific to OPN only [7].

It is important to investigate the etiology of OPN as it affects the management and treatment options. A comprehensive array of laboratory tests is necessary, encompassing serological tests for syphilis [8]; serum angiotensin-converting enzyme (ACE) levels for sarcoidosis [9]; Mantoux test along with chest X-ray for tuberculosis, as well as ANCA, IgG4, and erythrocyte sedimentation rate evaluations for conditions such as giant cell arteritis (GCA), granulomatosis with polyangiitis [10] and Behcet’s disease [11]. Lumbar puncture cerebrospinal fluid analysis is essential to exclude malignancy and infection within the central nervous system [3]. In a recent study, some cases that had been diagnosed with idiopathic optic perineuritis were found to be associated with neuromyelitis optica spectrum disorder (NMOSD) [6]. In the present case, the investigation for anti-aquaporin-4 antibodies was done in view of presence of periventricular hyperintense white matter lesion in MRI that could be due to NMOSD. However the result was negative.

OPN is generally progressive and blinding, with high-dose corticosteroids serving as the primary treatment approach [3,12]. Patients with OPN generally have a favourable prognosis with a rapid and dramatic improvement of visual recovery, typically within hours or within a day after the initiation of corticosteroid [3,12]. In order to prevent relapses and recurrent episodes of OPN, a subsequent longer course of oral corticosteroid therapy with gradual tapering of the dosage is necessary [2]. The investigations should be thorough to exclude the underlying cause of OPN and a specific treatment of such causes is necessary apart from corticosteroid therapy [1].

The ultimate visual prognosis of OPN is affected by delays in starting steroid therapy after the onset of visual impairment, the frequency of recurrent episodes, and the presence of associated systemic conditions [3,12]. It has been reported that OPN arising secondary to Behcet's disease is associated with a poor prognosis [11]. Prolonged administration of high-dose steroids in OPN can result in complications [3].

## Conclusions

Intravenous megadose corticosteroid treatment is an effective first-line treatment for optic perineuritis. Regardless of how severe the visual impairment at presentation, a good final visual outcome is possible. Cases with delayed treatment initiation have been associated with a poor prognosis. Therefore, early diagnosis and prompt treatment of OPN are crucial.
